# Attitudes of US Obstetricians Toward a Combined Tetanus-Diphtheria-Acellular Pertussis Vaccine for Adults

**DOI:** 10.1155/IDOG/2006/87040

**Published:** 2006-10-03

**Authors:** Sarah J. Clark, Soukaina Adolphe, Matthew M. Davis, Anne E. Cowan, Katrina Kretsinger

**Affiliations:** ^1^Child Health Evaluation and Research Unit, University of Michigan, Ann Arbor, MI 48109, USA; ^2^Baystate Medical Center, Springfield, MA 01199, USA; ^3^Division of General Internal Medicine, University of Michigan, Ann Arbor, MI 48109, USA; ^4^Gerald R. Ford School of Public Policy, University of Michigan, Ann Arbor, MI 48109, USA; ^5^National Center for Immunization and Respiratory Diseases, Centers for Disease Control and Prevention, Atlanta, GA 30333, USA; ^6^Commissioned Corps of the United States Public Health Service, USA

## Abstract

*Objective*. To describe obstetricians' perspectives related to tetanus-diphtheria-acellular pertussis (Tdap) vaccination of mothers and other adults in close contact with infants. *Methods*. Mail survey of national random sample of 400 obstetricians . *Results*. Response rate was 54%. Most respondents would likely recommend Tdap for women during the postpartum hospital stay (78%) or during pregnancy (69%) if a national recommendation was issued. Expected barriers were knowing the date of patients' most recent Td booster (74%) and patient resistance (46%). Most felt that obstetricians have a role in promoting and administering Tdap vaccine to adults other than mothers likely to come in close contact with infants. *Conclusion*. Obstetricians are likely to agree with the recent provisional US recommendation to administer Tdap to postpartum mothers and other adults expected to come in close contact with infants. Obstetricians would also be likely to support a potential recommendation to administer Tdap during pregnancy. Barriers to adoption of new Tdap vaccine recommendations should be monitored.

## INTRODUCTION

Pertussis is a highly communicable infection that carries substantial morbidity. The incidence of reported pertussis is increasing in all age groups, with over 25000 cases reported to the Centers for Disease Control and Prevention (CDC) in 2004 [1]. Infants, especially those who have not yet received the routine three-dose primary series of diphtheria-tetanus-pertussis vaccine, are particularly vulnerable to severe complications and death from pertussis [2], and often become infected through exposure to infected adults [3].

The ability to vaccinate adults against pertussis would reduce morbidity among adults and help prevent transmission to vulnerable infants. Prior to 2005, no pertussis vaccine was licensed in the United States for adults. In June 2005, the US Food and Drug Administration (FDA) approved licensure for a combination tetanus-diphtheria-acellular pertussis booster vaccine (Tdap, ADACEL, sanofi pasteur) for one-time use as a single dose in persons 11–64 years of age. Tdap has been shown to be safe and immunogenic in men and nonpregnant women; no controlled trials have been conducted to examine the safety and immunogenicity of Tdap during pregnancy or its effects on the infant immune response.

Following licensure, the US Advisory Committee on Immunization Practices (ACIP), with liaison representation from the American College of Obstetricians and Gynecologists (ACOG) and other partners, deliberated regarding recommendations for use of this new vaccine in adults. In particular, the ACIP discussed targeting a Tdap recommendation to women and other adults likely to come in close contact with infants.

Because obstetricians are an important direct link to women who are or will soon be in close contact with infants, we undertook a study to explore their perspectives regarding potential administration of Tdap to postpartum mothers or pregnant women, and to other adults expected to come in close contact with infants. These data were provided to the ACIP in October 2005.

## METHODS

### Sample

A national random sample of 400 obstetricians was drawn from the AMA Masterfile through an AMA-endorsed contracted vendor (Medical Marketing Service, Inc.). The AMA Masterfile, a database of all licensed US physicians, is the most comprehensive physician listing in the United States, and includes both AMA members and nonmembers. The sampling frame included all allopathic and osteopathic physicians self-described as an obstetrician/gynecologist, in office-based direct patient care. Excluded were physicians with any subspecialty board listing, physicians 70 years of age or older, resident physicians, and physicians practicing at federal government (ie, Veterans Affairs, military) medical facilities. The study was approved by the Institutional Review Board of the University of Michigan Medical School, with a waiver of documentation of informed consent.

### Survey instrument

The study team developed a one-page, 6-item survey instrument, accompanied by a one-page “Fact Sheet” regarding pertussis disease and the recently licensed Tdap vaccine. To verify eligibility, a screening item asked whether the respondent currently provides obstetric care. Survey items included current approach to administering influenza and measles-mumps-rubella (MMR) vaccines; likelihood of recommending Tdap vaccine during pregnancy and during the postpartum hospital stay, if recommended by ACIP/ACOG; barriers to administering Tdap vaccine to obstetric patients; and perceived responsibility for promoting or administering Tdap vaccine to adults expected to come in close contact with young infants.

The survey instrument and Fact Sheet were pilot tested with a convenience sample of obstetricians to ensure clarity and ease of administration. Refinements were made based on pilot test feedback.

### Survey administration

To meet the timeframe of the ACIP workgroup, only one mailing of the survey was fielded, in August 2005. Survey packets contained a cover letter explaining the purpose of the study, the Fact Sheet and survey form, and a $5 cash incentive.

### Data analysis

Initial univariate frequencies were generated for each variable. Chi-square analyses were performed to explore associations between variables. A two-tailed *α* level of .05 was used as the threshold for statistical significance. All analyses were conducted using SAS version 8.2 (SAS, Inc., Cary, NC).

## RESULTS

### Sample characteristics

Of the 400 obstetricians in the study sample, 5 were excluded because mailing materials were returned as undeliverable. Surveys were returned by 212 respondents, for an overall response rate of 54%. Of the 212 respondents, 29 were ineligible because they do not provide obstetric care, leaving 183 surveys eligible for analysis. With regard to demographic characteristics of this group, 79% are board-certified in obstetrics-gynecology; 40% are female; 43% are more than 50 years of age; and 47% work in a multispecialty practice site.

### Current approach to administering vaccines to obstetric patients

Most respondents (87%) reported that they *routinely* administer MMR vaccine to rubella nonimmune women immediately after delivery (postpartum hospital stay); 4% reported *sometimes*; and 9% reported *rarely/never* administering MMR vaccine. In comparison, 61% reported *routinely* administering influenza vaccine to pregnant patients; 19% reported *sometimes*; and 20% reported *rarely/never* administering influenza vaccine during pregnancy.

### Likelihood of recommending Tdap vaccine for obstetric patients

Overall, 78% of respondents *agree* or *strongly agree* that they would likely recommend Tdap vaccine for women immediately after delivery (in the postpartum hospital stay) if recommended by ACIP/ACOG. A lower proportion (69%) of respondents *agree* or *strongly agree* that they would likely recommend Tdap vaccine for women during pregnancy if recommended by ACIP/ACOG. For both questions, only 9% of respondents *strongly disagree* that they would recommend Tdap vaccine. Respondents' extent of agreement with these statements did not differ by their current approach to administering influenza vaccine or MMR vaccine.

### Anticipated barriers to Tdap vaccination of obstetric patients

Under the assumption of an ACIP/ACOG recommendation for Tdap vaccination of postpartum and/or pregnant women, the most commonly expected *major* barrier to vaccination was knowing the date of a patient's most recent Td booster (74% of respondents). Nearly half of respondents (46%) felt patient reluctance or refusal would be a *major* barrier, while 19% reported that having other priorities during obstetric visits would be a barrier. With regard to an open-ended question about other barriers, 14% of respondents noted cost-related issues (eg, reimbursement, vaccine cost) as a potential barrier.

In bivariate analyses, anticipated barriers cited by respondents were not associated with their likelihood of recommending Tdap vaccine to postpartum or pregnant women.

### Perspectives on Tdap vaccine for adults in close contact with infants

Respondents were asked which physician group(s) should bear responsibility for promoting and administering Tdap vaccine to adults likely to come in close contact with infants ≤ 6 months of age, assuming that an ACIP/ACOG recommendation would target this group. With regard to *promoting* Tdap vaccination, respondents perceived a shared responsibility among obstetricians (72%), adult primary care providers (81%), pediatricians (68%), and public health providers (60%). With regard to *administering* Tdap vaccine to adults likely to come in close contact with infants ≤ 6 months of age, respondents perceived a greater responsibility for adult primary care providers (89%) compared with obstetricians (62%), pediatricians (24%), and public health providers (61%). In bivariate analyses, those who were likely to recommend Tdap vaccine to postpartum or pregnant women, if recommended by ACIP/ACOG, were more likely to perceive themselves as having a role in both promoting and administering Tdap vaccine to other adults likely to come in close contact with infants (Table 1).

## DISCUSSION

In October 2005 the ACIP, with liaison representation from ACOG and other partners, voted to recommend that adults 19–64 years receive a single dose of Tdap vaccine to replace their next Td dose [4]. ACIP also voted to recommend that adults in close contact, or anticipating close contact, with an infant < 12 months of age (eg, parents, grandparents, childcare providers, and health care workers) receive a dose of Tdap as soon as feasible, if they have not previously received Tdap. The recommendation suggests an interval of 2 years since the most recent Td, although shorter intervals may be used. In addition, women planning a pregnancy and women in the immediate postpartum period are advised to receive Tdap if they have not previously done so. Pregnancy is not considered a contraindication to Tdap vaccination, and guidance on the use of Tdap during pregnancy was still under consideration by ACIP and ACOG.

Results of this study, conducted prior to these recommendations, demonstrate that obstetricians will likely support Tdap vaccination of obstetric patients if recommended by ACIP/ACOG. There appeared to be a slight preference for vaccination in the immediate postpartum period; still, the majority of respondents felt they would recommend vaccination during pregnancy, if endorsed in the future by ACIP/ACOG. This sentiment is consistent with prior research demonstrating that most obstetricians recommend influenza vaccination during pregnancy [5]. Note that the Fact Sheet included with this survey did state that there is no comprehensive information on the benefits and risks of Tdap vaccination during pregnancy, as is true for other recommended vaccines [6].

The factors influencing obstetricians' perceived likelihood of recommending Tdap to pregnant or postpartum women, if recommended by ACIP/ACOG, are unclear. While it might be assumed that obstetricians who routinely administer MMR and influenza vaccines would be more inclined to consider themselves likely to recommend Tdap vaccine, we did not find that to be true in this study. In addition, perceived barriers to Tdap administration were not associated with predicted likelihood of recommending Tdap. Prior research has shown ACOG endorsement to be an important influence on obstetrician decisions about vaccination [7]. It is likely that practicing obstetricians expect the members of ACIP and ACOG to craft the most appropriate Tdap recommendations after reviewing the available evidence.

Practice patterns for other vaccines can provide important clues to the uptake of new vaccines. Practices related to MMR vaccine for rubella nonimmune women in the postpartum period may provide the best analogy to the Tdap postpartum vaccination recommendation. In our study, 87% of obstetricians reported that they *routinely* administer MMR vaccine to postpartum mothers as indicated. However, logistical barriers to postpartum MMR vaccination remain; previous studies have shown that a significant proportion of obstetric practices do not stock MMR [7] and that a significant proportion of hospitals do not have rubella immunization programs for postpartum women [8]. Adoption of Tdap vaccine by obstetricians may be hampered by these same barriers.

This study identified several barriers to Tdap vaccination for obstetric patients. Three quarters of respondents felt that not knowing the date of their patient's most recent Td booster would be a major barrier to Tdap vaccination. This problem may be alleviated somewhat by the flexible timeframe for Tdap vaccination; the recommendation suggests an interval as short as 2 years since the last Td booster, and explicitly states that even shorter intervals may be used [4]. However, it is unclear if physicians will be comfortable with this abbreviated timeframe. Future clinical trial data is needed to address issues related to the minimum timeframe between Td and Tdap administration.

Although our survey did not directly ask about cost-related issues, 14% of respondents noted cost as a major barrier in an open-ended question on other barriers. Certainly, cost has been cited previously as a barrier to immunization, among both obstetricians [7] and primary care providers [9]. The extent to which cost will influence decisions related to stocking and administering Tdap vaccine is not yet clear. We cannot say from this study what proportion of obstetric practices currently stock Td vaccine, which may influence their decisions about the Tdap vaccine; Tdap costs about $20 more than Td per dose.

A majority of respondents to this survey felt that obstetricians should be involved in promoting Tdap vaccination for other adults expected to come in close contact with infants. This is entirely reasonable; during prenatal care, obstetricians have an opportunity to educate prospective parents about the importance of vaccination for grandparents, childcare providers, and other close contacts. We found that a slightly smaller proportion perceived a responsibility to administer Tdap vaccine, along with other provider groups. These results are consistent with prior research on other vaccines recommended for obstetric patients [7]. In practical terms, it is unrealistic to expect obstetricians to administer a vaccine to other adults who are not their patients, as there would be significant difficulties with billing and recordkeeping, as well as potential concerns regarding liability.

## LIMITATIONS

Studies utilizing mailed surveys have inherent limitations. Response bias may have affected our results. Based on the limited set of demographic variables available, the only difference between respondents and nonrespondents was that respondents were more likely to be board certified. In addition, we would expect that our sample is representative of all US obstetricians within our sampling frame (eg, providing direct patient care, no subspecialty board certification), given that we obtained a random sample from a national physician database.

We acknowledge that response bias likely exists, but it is impossible to detect its direction. While it is possible that those who responded to the survey were more interested in vaccination issues or had prior clinical experience with pertussis, the response rate is comparable to recently published results from other national, mailed surveys of US obstetricians, on both vaccination-related topics [5, 7] and a range of other obstetric issues [10–13]. In addition, the response rate is favorable compared to these other studies in that (1) we were limited to one mailing of the survey, to meet the timeframe for ACIP deliberations, and (2) it is higher than the response rates among those obstetricians who were not part of an established research network [5, 9, 10, 12].

## CONCLUSIONS

US obstetricians are likely to agree with the recent national recommendation to administer Tdap to postpartum mothers and other adults expected to come in close contact with vulnerable infants. In addition, they would support a recommendation to immunize pregnant women with Tdap, if recommended by ACIP/ACOG at a later date. However, future research is needed to assess the extent to which barriers will impede adoption of new Tdap vaccine recommendations.

## Figures and Tables

**Table 1:** Differences in obstetricians' perceived responsibility for promoting or administering Tdap vaccine to adults in close contact with infants (*P* value compares responses of agree/strongly agree versus neutral/disagree/strongly disagree).

	Proportion of respondents who agree that obstetricians have responsibility for			
	
	*Promoting* Tdap vaccine among		*Administering* Tdap vaccine to	
	adults in close contact with infants		adults in close contact with infants	

I would likely recommend Tdap vaccine
for women during pregnancy if
recommended by ACIP/ACOG
Agree/strongly agree	77%	< .001	75%	< .001
Neutral/disagree/strongly disagree	50%	—	60%	—

I would likely recommend Tdap vaccine
for women immediately after delivery if
recommended by ACIP/ACOG
Agree/strongly agree	85%	< .05	83%	< .05
Neutral/disagree/strongly disagree	59%	—	68%	—

## References

[1] Centers for Disease Control Prevention (CDC) (2005). Notice to readers: final 2004 reports of notifiable diseases. *Morbidity and Mortality Weekly Report*.

[2] Vitek CR, Pascual FB, Baughman AL, Murphy TV (2003). Increase in deaths from pertussis among young infants in
the United States in the 1990s. *Pediatric Infectious Disease Journal*.

[3] Bisgard KM, Pascual FB, Ehresmann KR (2004). Infant pertussis: who was the source?. *Pediatric Infectious Disease Journal*.

[4] ACIP votes to recommend use of combined tetanus,
diphtheria and pertussis (Tdap) vaccine for adults. http://www.cdc.gov/nip/vaccine/tdap/.

[5] Centers for Disease Control Prevention (CDC) (2005). Influenza vaccination in pregnancy: practices among
obstetrician-gynecologists—United States,
2003-2004 Influenza season. *Morbidity and Mortality Weekly Report*.

[6] Centers for Disease Control Prevention (CDC) Guidelines for vaccinating pregnant women. http://www.cdc.gov/nip/publications/.

[7] Schrag SJ, Fiore AE, Gonik B (2003). Vaccination and perinatal infection prevention practices
among obstetrician-gynecologists. *Obstetrics and Gynecology*.

[8] Bath SK, Singleton JA, Strikas RA, Stevenson JM, McDonald LL, Williams WW (2000). Performance of US hospitals on recommended screening and
immunization practices for pregnant and postpartum women. *American Journal of Infection Control*.

[9] Davis MM, Ndiaye SM, Freed GL, Kim CS, Clark SJ (2003). Influence of insurance status and vaccine cost on
physicians' administration of pneumococcal conjugate vaccine. *Pediatrics*.

[10] Chamany S, Schulkin J, Rose CE, Riley LE, Besser RE (2005). Knowledge, attitudes, and reported practices among
obstetrician-gynecologists in the USA regarding antibiotic
prescribing for upper respiratory tract infections. *Infectious Disease in Obstetrics and Gynecology*.

[11] Cleary-Goldman J, Morgan MA, Malone FD, Robinson JN, D'Alton ME, Schulkin J (2006). Screening for Down syndrome: practice patterns and knowledge
of obstetricians and gynecologists. *Obstetrics and Gynecology*.

[12] Coleman VH, Erickson K, Schulkin J, Zinberg S, Sachs BP (2005). Vaginal birth after cesarean delivery: practice patterns of
obstetrician-gynecologists. *Journal of Reproductive Medicine for the Obstetrician
and Gynecologist*.

[13] Hankins GDV, Erickson K, Zinberg S, Schulkin J (2003). Neonatal encephalopathy and cerebral palsy: a knowledge
survey of Fellows of The American College of Obstetricians
and Gynecologists. *Obstetrics and Gynecology*.

